# Lipid profile modifications of the lung tissue and surfactant in a murine model of malaria associated ARDS

**DOI:** 10.1186/1475-2875-11-S1-P87

**Published:** 2012-10-15

**Authors:** Diletta Scaccabarozzi, Natacha Lays, Lucia Cortelezzi, Philippe Van den Steen, Ghislain Opdenakker, Donatella Taramelli, Fausta Omodeo-Salè

**Affiliations:** 1 Dipartimento di Scienze farmacologiche e Biomolecolari, Università degli studi di Milano, Milan, 20134, Italy; 2 Rega Institute for Medical Research, Catholic University of Leuven, Leuven, 3000, Belgium

## Background

One of the lethal complications of malaria is acute lung injury and in its more severe form, acute respiratory distress syndrome (ARDS). In the murine model of malaria-associated ARDS (C57BL/6 infected with *Plasmodium berghei pb*NK65) the cytokine profile of infected animals is significantly altered in relation to the percent parasitemia. Preliminary data from our laboratory have shown alterations of the fatty acid profile in lungs of mice infected by *pb*NK65. However, it is not known if the molecular organization and lipid composition of pulmonary surfactant change during malaria ARDS. Surfactant once secreted forms organized lipid structures referred as *large aggregates* (LA). During respiration an inactive form of surfactant is also produced, the *small aggregate* form (SA). We explored the lipid profile both of the aggregates and of the lung tissue from non infected and infected animals with *pb*NK65 and with *Plasmodium chabaudi* (*PcAS*), a Plasmodium strain that does not induce lung pathology.

## Materials and methods

C57BL/6 mice were infected by *pb*NK65 or *Pc*AS parasites and sacrificed 6, 8 and 10 days after infection. Cell-free BAL was centrifuged to obtain the LA and SA fractions. The right lung was perfused and homogenate. PL content was quantified according to Bartlett and PL pattern by HPLTC or HPLC analysis.

## Results

An increase in the total content of PL of BAL in all the infected groups from day 8 post infection and increased levels of total proteins from days 6, were observed. Unexpectedly, the percentage of LA is significantly increased in NK65 mice as well the protein content and protein/PL ratio. These alterations are absent in the AS groups. The LA fraction of the NK65 shows a significant increase in the relative amounts of sphingomyeline and decrease of phosphatidilglycerol. The same changes were observed in the SA fraction accompanied by significant increase of lysophosphatidylcholine (LPC). The membrane enriched fractions of the lungs from NK65 mice are characterized by a significant increase of phosphatidylcholine and phosphatidylethanolamine. No differences are present in the other classes of PL and in the AS group.

## Conclusions

The increase in PL in the lung tissue is a common response to alveolar inflammation. This modification, absent in AS mice, appears to be correlated with malaria ARDS and consistent with the eosinophilic hyaline membrane deposition and cell infiltration observed in the alveoli of NK65 mice. The BAL fluid of NK65 mice is characterized by high increase of protein levels indicative of oedema and alveolar leakage due to the lung pathology. On the contrary, the total PL increase present also in AS groups, seems related to malaria infection but not to lung pathology. Increased total protein levels are present in the LA fraction of NK65 mice, probably due to blood-derived proteins being incorporated into or associated with these microstructures in the alveolar hypophase. The increase of LPC, a known inhibitor of surfactant activity, in the SA fraction of NK65 mice is consistent with the action of phospholipases which are known to be present in the lungs during inflammatory injury.

